# The role of immune checkpoint inhibitors in clinical practice: an analysis of the treatment patterns, survival and toxicity rates by sex

**DOI:** 10.1007/s00432-022-04309-2

**Published:** 2022-08-23

**Authors:** Murielle N. Wahli, Stefanie Hayoz, Dennis Hoch, Christoph O. Ryser, Michèle Hoffmann, Amina Scherz, Birgit Schwacha-Eipper, Simon Häfliger, Julian Wampfler, Martin D. Berger, Urban Novak, Berna C. Özdemir

**Affiliations:** 1 Department of Medical Oncology, Inselspital Bern, Bern University Hospital, University of Bern, Freiburgstrasse 41, 3011 Bern, Switzerland; 2 Swiss Group for Clinical Cancer Research, Bern, Switzerland; 3 Hepatology, Department of Biomedical Research, Inselspital Bern, Bern University Hospital, University of Bern, Bern, Switzerland; 4 University Clinic for Visceral Surgery and Medicine, Inselspital Bern, Bern University Hospital, University of Bern, Bern, Switzerland

**Keywords:** Immune checkpoint inhibitors, Role, Age, Sex, Gender, Real-world, Clinical practice

## Abstract

**Purpose:**

Our aim is to describe the role of immune checkpoint inhibitors (ICI) in clinical practice by providing the patient and tumor characteristics as well as survival and toxicity rates by sex.

**Methods:**

We used electronic health records to identify patients treated at the Cancer Center of the University Hospital Bern, Switzerland between January 1, 2017 and June 16, 2021.

**Results:**

We identified 5109 patients, 689 of whom (13.5%) received at least one dose of ICI. The fraction of patients who were prescribed ICI increased from 8.6% in 2017 to 22.9% in 2021. ICI represented 13.2% of the anticancer treatments in 2017 and increased to 28.2% in 2021. The majority of patients were male (68.7%), who were older than the female patients (median age 67 vs. 61 years). Over time, adjuvant and first line treatments increased for both sexes. Lung cancer and melanoma were the most common cancer types in males and females. The incidence of irAEs was higher among females (38.4% vs. 28.1%) and lead more often to treatment discontination in females than in males (21.1% vs. 16.8%). Independent of sex, the occurrence of irAEs was associated with greater median overall survival (OS, not reached vs. 1.1 years). Female patients had a longer median OS than males (1.9 vs. 1.5 years).

**Conclusions:**

ICI play an increasingly important role in oncology. irAEs are more frequent in female patients and are associated with a longer OS. More research is needed to understand the association between patient sex and toxicity and survival.

## Introduction

The development of immune checkpoint inhibitors (ICIs) is one of the greatest achievements of the last decade and has profoundly changed the treatment landscape for several cancer types. Currently, 7 antibodies targeting the programmed death 1 (PD-1) or its ligand (PD-L1) and one antibody targeting the Cytotoxic T-lymphocyte associated protein 4 (CTLA4) are FDA approved for over 85 indications (Beaver and Pazdur 2021). About 5000 clinical trials are actively testing ICI alone or in combination with other approved therapies or new compounds in different disease settings (Upadhaya et al. 2022).

The unprecedented success of this treatment approach (including the potential for long-term remission in a small subset of patients) often fuels the expectations from both patients and physicians, sometimes in a unjustified fashion (The Lancet 2018). Although the benefits from ICI are obvious in certain cancer types (e.g., melanoma), there is some controversy regarding the therapeutic and economic value of ICI in other indications given that several approvals were based on single arm trials and surrogate endpoints (e.g., response rate and progression free survival [PFS]) which correlate rather poorly with overall survival (OS) (Hilal et al. 2020; Haslam et al. 2019; Prasad et al. 2015). In addition, immune-related adverse events (irAEs) can occur at any time, including after terminating ICI treatment. IrAEs can affect all tissues and be irreversible and sometimes require long-term corticosteroid treatment or substitution of various hormonal axes (Ghisoni et al. 2021). The investigation of a potential negative effect of ICI on fertility is also largely missing, although the available limited evidence indicate that impaired spermatogenesis might be an issue in some men treated with ICI (Ozdemir 2021).

Importantly, patients receiving ICI in a real-world setting may significantly differ from those enrolled in clinical trials, including in factors related to age, performance status, and comorbidities. Although an analysis of the US population indicates that the percentage of cancer patients who are eligible for ICI has substantially increased from 1.5% in 2011 to 43% in 2018 (Haslam and Prasad 2019), a large fraction of the patients in clinical practice are not represented in trials given their strict selection criteria. Recent studies found that 55% of patients with metastatic melanoma (Donia et al. 2017) and 70% of patients with non-small-cell lung cancer (NSCLC) (Yoo et al. 2018) would not fulfill eligibility criteria for phase 3 trials testing ICIs, which raises issues regarding the likely limited generalizability of the trial findings to the real-world setting. In line with this, in a large analysis of US patients receiving first line therapies for advanced NSCLC, the median OS for pembrolizumab monotherapy or pembrolizumab with chemotherapy was 15 months and 10 months, respectively, which was shorter than that reported in the Keynote 024 trial (Kehl et al. 2021). Similarly, a multicenter analysis from the Netherlands reported comparable PFS times yet shorter OS times for first- or second-line ICI for NSCLC, possibly related to the finding that fewer patients received subsequent lines of treatment in real-world settings as compared to trials (Cramer-van der Welle et al. 2021). In addition, the outcome of older, frail patients seems to be significantly poorer than those of the patients included in clinical trials (La et al. 2020). In addition, the potential impact of a patients’ (biological) sex and (socio-cultural) gender on treatment patterns, toxicity and efficacy is gaining increasing attention in oncology and more research is needed to provide insights into disparities between male and female patients (Ozdemir et al. 2022).

Despite the increasing number of reports on real-world outcomes of ICI for specific cancer types, analyses defining the role of ICI in oncology practice are largely missing. The aim of our real-world analysis is to depict the role of ICI treatments in a European University Cancer Center by assessing the percentage of ICI treatments among all systemic anticancer treatments prescribed, characterizing the patient population as well as tumor and treatment types and duration, and providing OS and toxicity rates by sex.

## Methods

### Study population

Medical records from the Bern University Hospital Cancer Center were used to identify patients with the following eligibility criteria: aged 18 years or older, and prescribed at least one cycle of ICI therapy between January 1, 2017 and June 16, 2021. Information about each identified patient was documented including: biological sex; age at treatment start; cancer type; disease stage; presence of brain metastasis at diagnosis; type of ICI administered; date of initiation of ICI therapy; line of ICI, number of ICI treatment cycles; radiological response to treatment; duration of treatment; reason for treatment discontinuation; any grade irAEs, treatment with immunosuppressive drugs for irAEs, presence of preexisting autoimmune or neurological disorders (e.g., dementia, epilepsy without brain metastases) or chronic infections (e.g., HIV, Hepatitis B and C) or hematological malignancies. Treatment doses and schedules were as follows: 3 mg/kg or 200 mg of pembrolizumab every 3 weeks; 3 mg/kg or 240 mg of nivolumab every 2 weeks; 350 mg of cemiplimab every 3 weeks; 1200 mg of atezolizumab every 3 weeks; 10 mg/kg of durvalumab every 2 weeks; 10 mg/kg of avelumab every 2 weeks; 3 mg/kg or 1 mg/kg of ipilimumab every 3 or 6 weeks, depending on the indication.

Maintenance treatment was defined as continuation of single agent ICI after an initial combination treatment. The data cutoff date was October 31, 2021.

### Statistical analysis

Descriptive statistics were used to describe patient, tumor, and treatment characteristics. Median OS was estimated for each subgroup using the Kaplan–Meier method and illustrated using Kaplan–Meier curves. Patients who did not have an event (i.e., death) by the date of treatment termination, and patients with ongoing treatment were censored at the last follow-up. The median follow-up duration was calculated with the reversed Kaplan–Meier method.

Statistical analyses were performed using R Version 4.2.0.

## Results

### The role of ICI in total treatment population

We identified a total of 5109 unique patients who were prescribed one or more systemic anticancer treatment, including cytotoxic drugs, targeted therapies, and cellular therapies at our center. ICI were prescribed to 820 patients (16.0%), 97 of whom were excluded from analysis due to incomplete data (*n* = 36) or of lack of consent to use their data (*n* = 61). Of the remaining 723 ICI-prescribed patients (14.1%) who were eligible for analysis, 34 patients did not receive any ICI treatment either because of poor performance status or death prior to treatment initiation (*n* = 19), transfer to an external hospital (*n* = 3), or because they received a placebo in a clinical trial (*n* = 12). After excluding these individuals, our study included 689 patients who received at least one dose of ICI (corresponding to 13.5% of the total patient population who received anticancer treatment during the indicated time period).

The fraction of patients who were prescribed ICI increased from 8.7% in 2017 to 22.9% in 2021. Several patients (*n* = 109) received more than one line of ICI treatment, ICI treatments comprised 13.2% of all anticancer treatments in 2017 and increased to 28.3% in 2021 (Table 1).

**Table 1:** Prescription patterns of ICI treatments at the Bern University Hospital Cancer Centre between 2017 and 2021

	2017	2018	2019	2020	2021
Number of all anticancer treatments	7368	8846	11,038	11,238	5816
Number of ICI treatments	973	1736	2479	2995	1644
% of ICI of total treatment	13.2	19.6	22.5	26.7	28.3
Number of patients who were prescribed an anticancer treatment	1430	1579	1942	1979	1421
Number of patients who were prescribed an ICI treatment	124	188	321	372	326
% of ICI patients of total patients treated	8.7	11.9	16.5	18.8	22.9

Patients who continued treatment over the years were counted repeatedly

### Sex and age distribution

The majority of patients were male (68.7%; *n* = 473). The median age at the time of ICI treatment initiation was 66 years (range = 22–89 years) and varied by sex, with male and female patients having a median age of 67 years (range = 23–88 years) and 61 years (range = 21–87 years) at treatment start, respectively.

We observed differences in age distribution by sex, with 23% the female patients being younger than 50 years and 63.6% younger than 65 years as compared to 6.8 and 43.2% of male patients, respectively. 27.6% of the females and 38.1% of the males, respectively, were between 66–75 years. While 18.8% of the male patients were aged over 75 years, this age category represented only 9.2% of the female population.

### Comorbidities

We noted the presence of selected comorbidities which might affect treatment prescriptions, such as preexisting autoimmune diseases including Hashimoto thyroiditis (9.4%, *n* = 65), hematological malignancies (2.6%, *n* = 18), dementia (1%, *n* = 7), epilepsy without brain metastases (1.3%, *n* = 9), hepatitis B or C (3.9%, *n* = 27), and human immune deficiency virus (HIV, 0.4%, *n* = 3). The distribution of the comorbidities was similar between female and male patients.

### Tumor type, stage and PD-L1 expression

For both sexes, NSCLC was the most common cancer type followed by melanoma and urothelial cell carcinoma. With the exception of hepatocellular carcinoma (HCC, 9.9% vs. 2.8%) and mesothelioma (2.5% vs. 0.5%) which were considerably more common in male compared to female patients, most non-sex-related cancer types were represented at a similar frequency. Other cancer types included rare diseases, such as Merkel cell carcinoma, mycosis fungoides, neuroendocrine tumors, testicular chorion carcinoma and rare subtypes of lung cancer, or diseases without ICI approval, such as pancreas cancer and cholangiocarcinoma. PD-L1 status was known for 52.8 and 43.8% of the tumors in female and male patients, respectively (Table 2).

**Table 2:** Characteristics of the patients, their tumor, ICI treatment and irAEs by sex

	Male patients	Female patients
Patients receiving ICI, *n* = 689	473 (68.7%)	216 (31.3%)
Age (years)		
Median (range)	67 (23–88)	61 (21–87)
Cancer type		
NSCLC	154 (32.6%)	66 (30.6%)
Melanoma	86 (18.2%)	47 (21.8%)
Urothelial carcinoma	50 (10.6%)	16 (7.4%)
Hepatocellular carcinoma	47 (9.9%)	6 (2.8%)
Renal cell carcinoma	31 (6.6%)	13 (6.0%)
HNSCC	30 (6.3%)	11 (5.1%)
SCLC	20 (4.2%)	15 (6.9%)
Gastrointestinal cancers	18 (3.8%)	5 (2.3%)
Triple negative breast cancer		16 (7.4%)
Mesothelioma	12 (2.5%)	1 (0.5%)
Gynecological cancers		9 (4.2%)
Other	25 (5.3%)	11 (5.1%)
*Tumor stage*		
AJCC 8th edition staging		
I	7 (1.5%)	0
II	14 (3.0%)	8 (3.7%)
III	79 (16.7%)	43 (19.9%)
IV	307 (64.9%)	144 (66.7%)
SCLC staging		
Limited disease	1 (0.2%)	1 (0.5%)
Extensive disease	19 (4.0%)	14 (6.5%)
HCC BCLC staging		
BCLC A	9 (1.9%)	0
BCLC B	15 (3.2%)	2 (0.9%)
BCLC C	21 (4.4%)	4 (1.9%)
BCLC D	1 (0.2%)	0
PD-L1 status		
Known	207 (43.8%)	114 (52.8%)
Unknown	266 (56.2%)	102 (47.2%)
Total number of treatments, *n* = 798	541 (67.8%)	257 (32.2%)
*Treatments received*		
Anti-PD1		
Nivolumab	160 (29.6%)	63 (24.5%)
Pembrolizumab	135 (25.0%)	79 (30.7%)
Pembrolizumab + Chemotherapy	51 (9.4%)	24 (9.3%)
Pembrolizumab + Tyrosine kinase inhibitor	8 (1.5%)	4 (1.6%)
Cemiplimab	9 (1.7%)	1 (0.4%)
Anti-PD-L1		
Atezolizumab	17 (3.1%)	30 (11.7%)
Atezolizumab + Bevacizumab	46 (8.5%)	6 (2.3%)
Durvalumab	42 (7.8%)	11 (4.3%)
Avelumab	6 (1.1%)	1 (0.4%)
Anti-CTLA4		
Ipilimumab	5 (0.9%)	1 (0.4%)
Anti-CTLA4 + Anti-PD1		
Ipilimumab + Nivolumab	62 (11.5%)	37 (14.4%)
Number of cycles received (median, range), total	6 (1–102), 6160	6 (1–101), 2678
Anti-PD1		
Pembrolizumab	6 (1–61)	8 (1–74)
Nivolumab	9 (1–102)	8 (1–101)
Cemiplimab	10 (1–30)	49 (one treatm.)
Anti-PD-L1		
Atezolizumab	4 (1–20)	7 (1–23)
Durvalumab	10 (1–27)	9 (2–26)
Avelumab	11 (5–62)	23 (one treatm.)
Anti-CTLA4		
Ipilimumab	2 (2–4)	4 (one treatm.)
Anti-CTLA4 + Anti-PD1		
Ipilimumab + Nivolumab	3 (1–4)	3 (1–4)
Nivolumab maintenance	16 (1–76)	11 (1–82)
Treatment line		
Neoadjuvant	6 (1.1%)	10 (3.9%)
Neoadjuvant + Adjuvant	7 (1.3%)	2 (0.8%)
Adjuvant	67 (12.4%)	40 (15.6%)
First line	252 (46.6%)	121 (47.1%)
Second line	124 (22.9%)	44 (17.1%)
Third and further line	37 (6.8%)	18 (7.0%)
Maintenance	48 (8.9%)	22 (8.6%)
Treatment ongoing	71 (70.2%)	30 (29.7%)
Stable disease	41 (57.7%)	14 (46.7%)
Partial response	14 (19.7%)	13 (43.3%)
Complete response	11 (15.5%)	1 (3.3%)
Progressive disease	5 (7.0%)	2 (6.7%)
Immune-related adverse events (irAEs), *n* = 216		
Patients experiencing all grade irAEs	133 (28.1%)	83 (38.4%)
Patients experiencing 1 irAEs type	85 (63.9%)	54 (65.1%)
Patients experiencing 2 irAEs types	35 (26.3%)	21 (25.3%)
Patients experiencing 3 irAEs types	11 (8.3%)	8 (8.4%)
Patients experiencing > 3 irAEs types	2 (1.5%)	0
Total number of irAEs, *n* = 322	202 (62.7%)	120 (37.2%)
Days until irAEs (median, range)	85 (1–1211)	91 (1–580)
Implication of organ specialist (% of patients)	88 (66.2%)	45 (54.2%)

**Fig. 1:**
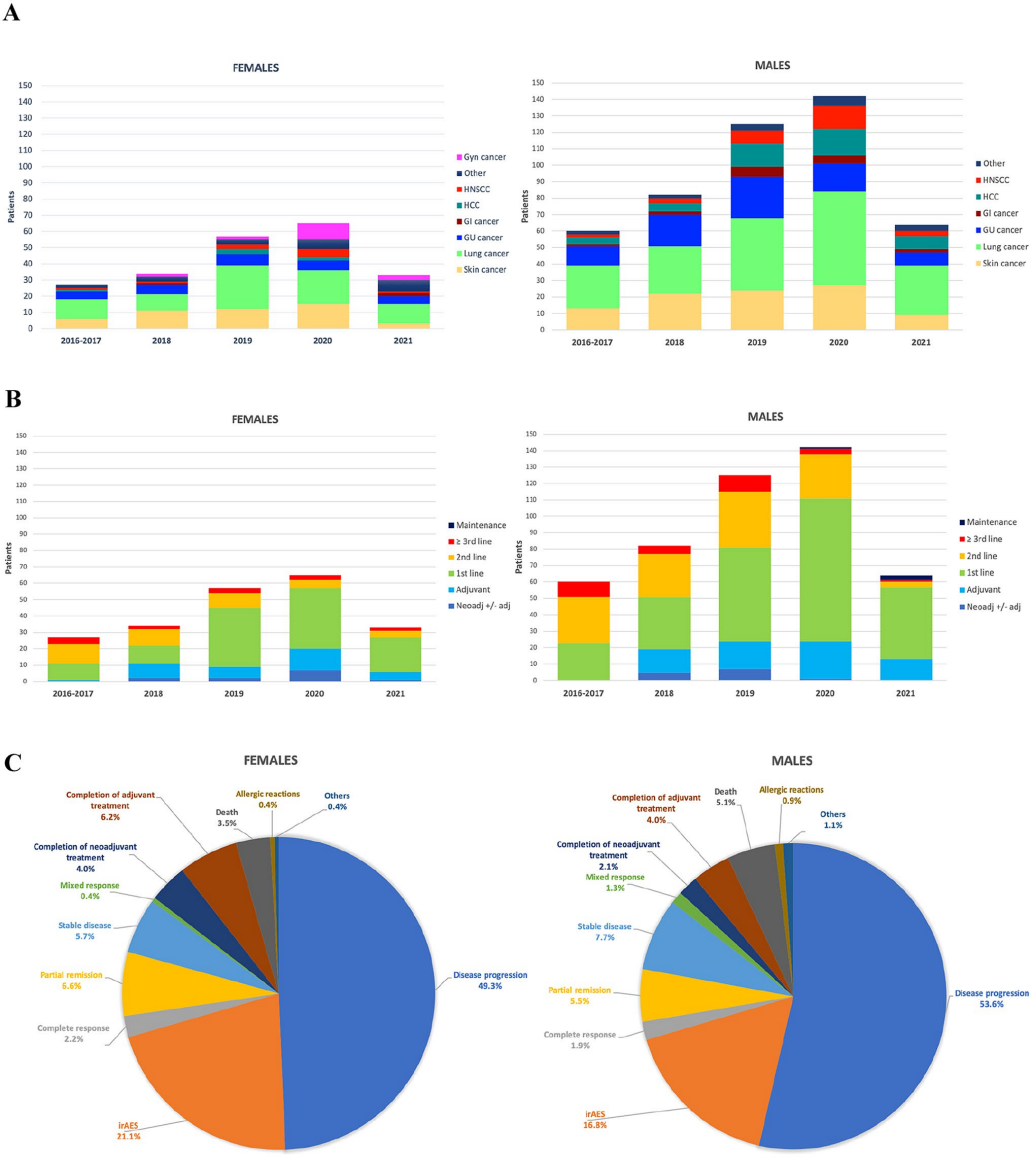
**A** Distribution of the tumor types over the years for female and male patients. **B** Distribution of the treatment settings over the years for female and male patients. **C** Reason for treatment discontinuation for female and male patients

The distribution of the cancer types changed during the years for both patient populations, with an increase in genitourinary (GU) and gastrointestinal (GI) cancers as well as HCC and head and neck squamous cell carcinomas (HNSCC) as a consequence of the rise in ICI approvals for these disease types (Fig. 1A).

### Treatment type, line and duration

Of the 798 ICI treatments, the vast majority were anti-PD1-based therapies. For male patients, nivolumab was the most frequent treatment type (29.6%), followed by pembrolizumab as monotherapy (25.0%), or in combination with chemotherapy or tyrosine kinase inhibitors (TKI) in 9.4 and 1.5% of the cases, respectively. For female patients, pembrolizumab monotherapy was the most frequent treatment (30.7%), the combination rates with chemotherapy (9.3%) or TKI (1.6%) were nearly identical as for males. Atezolizumab monotherapy was more often prescribed in females (11.7 vs. 3.1%), mostly for TNBC, while the combination with bevacizumab, as indicated for HCC, was more often prescribed in males (8.5% vs. 2.3%).

Around eight percent (7.8%, *n* = 62) of the ICI treatments, for 22 female and 40 male patients, were administered in the context of a clinical trial.

Independent of sex, ICI were most often prescribed for metastatic cancer (66.7% vs. 64.9%), as first line (47.1% vs. 46.6%) or second-line treatment (17.1% vs. 21.1%). Female patients had higher rates of adjuvant (15.6% vs. 12.4%) and neoadjuvant (3.9% vs. 1.1%) treatments. The rate of third or further line (7.0% vs. 6.9%) or maintenance treatments (8.6% vs. 8.9%) was very similar between male and female patients. The median number of treatment cycles did not show major differences between males and females. Over the years, for both sexes the fraction of adjuvant and first line treatments increased as compared to second and further line treatments (Table 2, Fig. 1B).

At the end of the study period, 101 treatments were ongoing (12.7%). The majority of the responses for the ongoing treatments in female and male patients were stable disease (SD 46.7% vs. 57.7%). Partial responses (PR) were found in 43.3% of the females as compared to 19.7% of the males. Complete response (CR) was more frequent in male (15.5%) than in female patients (3.3%). A similar fraction of female and male patients (6.7% vs. 7.0%) were treated beyond progressive disease (PD) (Table 2).

The main reasons for treatment discontinuation (*n* = 697) was PD in 49.3% of the female and 53.6% of the male patients. IrAEs led more often to discontinuation in females (21.1%) as compared to males (16.8%). The different reasons for treatment discontinuation for both sexes are depicted in (Fig. 1C).

### irAEs

Thirty one percent of the patients (31.3%, *n* = 216) experienced at least one type of irAE, and a total of 322 irAEs were reported for the duration of the 798 treatments.

The time to occurrence of irAEs (e.g., the time from treatment start to detection of the first irAE in cases of multiple irAEs) was a median of 85 days (1–1212 days). The vast majority of the irAEs were reported in the first 180 days (77.3%), 58.1% in the first 90 days and 23.1% in the first 30 days of treatment start. A small fraction of the irAEs were reported after the first 365 days (9.6%). Regarding the management of irAEs, for 61.5% (*n* = 133) of the patients, an organ specialist was consulted. Aside from corticosteroids, 27 (8.3%) of the 322 irAEs were treated with additional immunosuppressive agents (e.g., mycophenolat mofetil, azathioprin, methotrexat, infliximab, sulfasalazin, rituximab and sarilumab). Importantly, 38.8% (*n* = 84) of the patients received long-term hormonal substitution for ICI-related hypothyroidism (*n* = 64), hypophysitis (*n* = 14) or adrenal insufficiency (*n* = 6).

The age distribution of irAEs was heterogeneous. Over half (55.6%, *n* = 120) of the patients experiencing irAEs were ≤ 65 years, as compared to 28.1% (*n* = 61) of the affected patients who were 66–75 years and 16.2% of the patients who were older than 75 years.

The incidence of irAEs was higher in female patients (*n* = 83, 38.4%) compared to males (*n* = 133, 28.1%). The percentage of patients experiencing one or more types of irAEs was similar between males and females. The median time to occurrence of irAEs was 91 days (range = 1–580) for female patients and 85 days (range = 1–1212) for male patients. An organ specialist was implicated in the management of irAEs in 66.2% (*n* = 88) of the male patients as compared to 54.2% (*n* = 45) of the female patients (Table 2).

The frequency of the different irAEs was similar between females and males, the most common irAE was thyroiditis (23.3% vs. 20.3%), followed by colitis (15.8% vs. 16.3%) hepatitis (15.8% vs. 13.9%) and pneumonitis (7.5% vs. 12.4%). Skeletal adverse events (e.g., arthralgia, arthritis, tendinitis and spondylitis) were observed in 10.0% of the female and 7.9% of the male patients. Various skin reactions occurred at a similar frequency in females and males (7.5% vs. 6.4%).

**Fig. 2:**
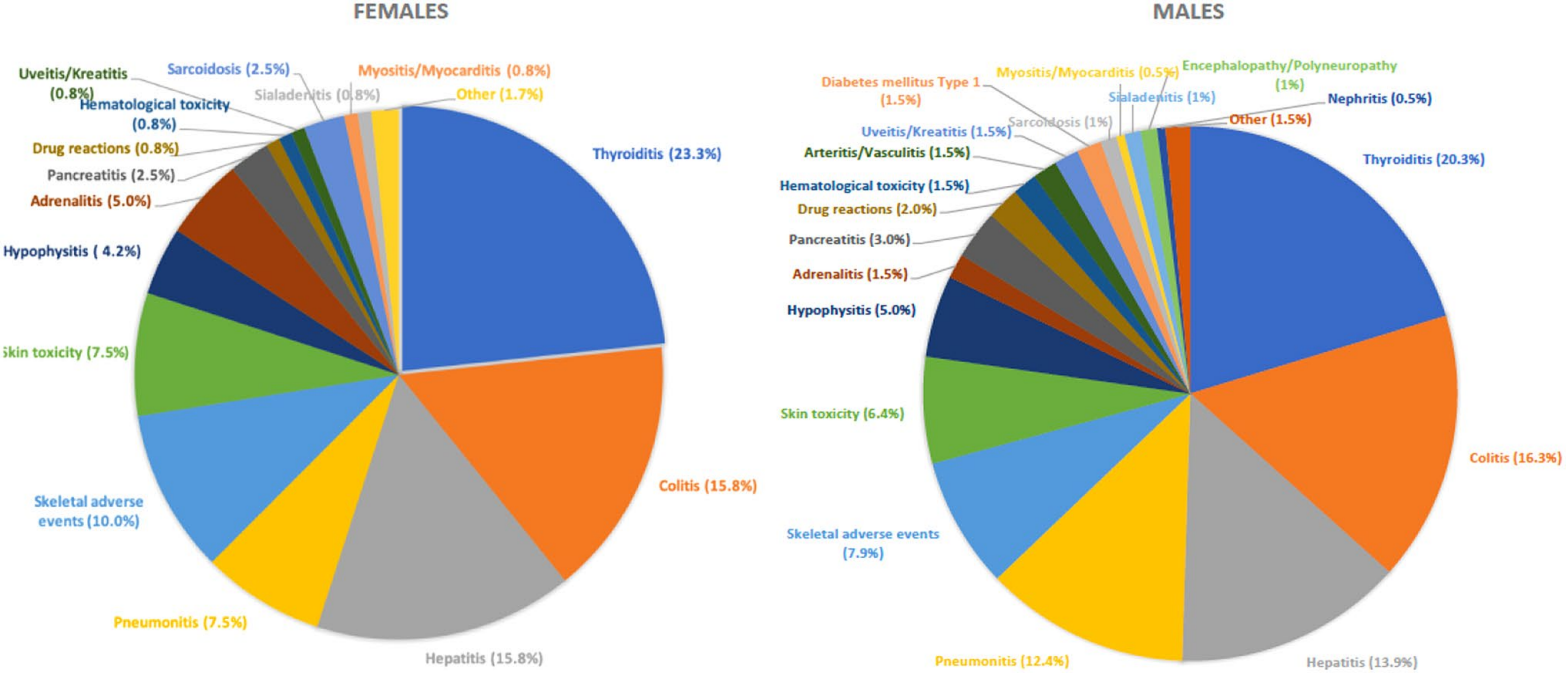
Type of all grade toxicity during or after treatment with ICI for women and men. More than one toxicity type per patient is depicted

The different types of irAEs are depicted in (Fig. 2).

### Overall survival

**Fig. 3:**
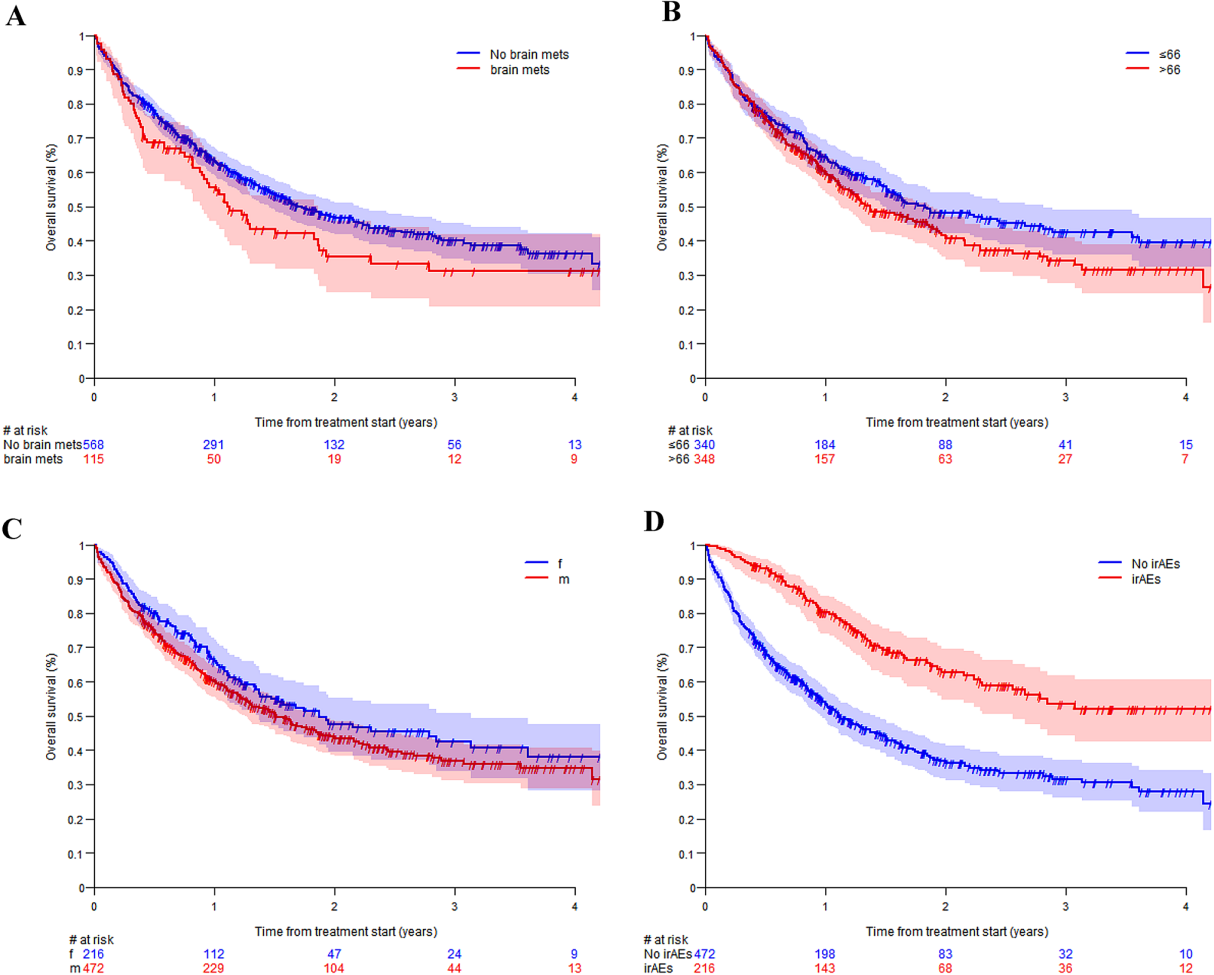
OS depicted for **A** presence of brain metastases at diagnosis, **B** median age, and **C** sex of the patients, and **D** any grade irAEs

The median follow-up time was 2.0 years (95% CI = 1.8–2.2). The median OS was 1.6 years (95% CI = 1.4–1.9). About seventeen percent (16.6%, *n* = 115), 36 female and 79 male patients, had brain metastases at diagnosis which was associated with a shorter OS (median = 1.1 years, 95% CI = 0.9–1.8) compared to those without (median = 1.7 years, 95% CI = 1.4–2.2) (Fig. 3A).

We analyzed the OS of the patients according to age, using the median age of 66 years as threshold. The curves separated at approximately 6 months and the OS was higher for patients ≤ 66 years (median = 1.8 years, 95% CI = 1.5–2.8) as compared to those > 66 years of age (median = 1.4 years, 95% CI = 1.1–1.9) (Fig. 3B).

OS was higher for female patients (median = 1.9 years, 95% CI = 1.4–3.1) compared to male patients (median = 1.5 years, CI = 1.3–1.8) (Fig. 3C). The OS advantage of the female population persisted across different age groups. Female patients ≤ 65 years of age had a median OS of 3.6 years (95% CI = 1.5-NR), as compared to 1.6 years (95% CI = 1.3–2.5) for males of the same age group. For female patients aged 66–75 years, the median OS was 1.4 (95% CI = 1.1–2.8) as compared to 1.3 (95% CI = 1.0–1.9) in the corresponding male population. Among those older than 75 years of age, male patients had a slightly longer median OS (1.9 years, 95% CI = 0.9-NR versus 1.6 years, 95% CI = 0.5–3.1). The incidence of irAEs was associated with a higher OS. For patients experiencing any type and grade of irAEs the median OS was not reached (95% CI = 2.6-NR) compared to 1.1 years (95% CI = 1.0–1.4) for those who did not report any irAEs (Fig. 3D).

## Discussion

Although an increasing number of reports on real-world outcomes of specific cancer types, such as lung cancer or melanoma exist, to our knowledge this is the first analysis that assesses the overall role of ICI in routine clinical practice at a European Cancer Center. Our findings indicate that the prescription rate of ICI as well as the fraction of patients receiving ICI has substantially increased over the past 5 years. The proportion of ICI treatments doubled in the same time period from 13.2% to 28.2%. This increasing share of ICI in clinical practice is in line with the expansion of approved ICI treatment indications and the prolonged duration of response in a subset of the patients who remained on treatment for an extended period, in some cases over several years.

In our center 7.8% of the ICI treatments were applied in the context of clinical trials. This is very similar to the contemporary clinical trial participation rates of 7.7–8.1% in the US (Unger et al. 2019; Byrne et al. 2014) and 7.7% in France (Ousseine 2022). Importantly, advanced age did not seem to be a limitation for ICI prescription, since 27.8% of the patients were > 75 years at time of treatment prescription. This finding is not surprising given that ICI are generally better tolerated and have lower discontinuation and mortality rates due to toxicity than chemotherapies, as shown in a large meta-analysis (Magee et al. 2020). In addition, a small fraction of the patients (1%) presented with preexisting dementia, which did not exclude them from receiving ICI and highlights the importance of individualized treatment decisions in patients with severe comorbidities.

The sex and age distribution of our population was comparable with previous reports (Waterhouse et al. 2021; Liu et al. 2019; Ghisoni et al. 2021). The male predominance may be attributed to the generally higher incidence of cancers in men (Haupt et al. 2021). The younger age of the female population (23% being younger than 50 years compared to 6.7% of the male patients) was possibly related to the large fraction of melanoma patients, given the universally higher rate of melanoma at a younger age in women (Yuan et al. 2019).

In agreement with the approved treatment indications, anti-PD1-based treatments were the predominant treatment type in female and male patients and only 12% of the treatments were ICI combinations. In our cohort the median number of ipilimumab and nivolumab induction cycles was three, independent of patient sex. This is in line with the report from the North American expanded access program for this combination (Checkmate 218), where 42, 24, 20, and 14% of the patients received 4, 3, 2, and 1 cycles, respectively (Hodi et al. 2021).

In our cohort, in both, female and male patients, disease progression (49.3% vs. 53.6) was the main reason for treatment discontinuation. Notably, independent of sex, only around 2% of the patients discontinuing treatment had a CR, the distribution of PR and SD was also similar between both patient populations. Interestingly, death of the patient was the reason for treatment discontinuation in 3.5% of the female and 5.1% of the male patients. It is conceivable that several of these patients presented rapid disease progression and death before a change in treatment type could be discussed and/or documented in the electronic health records.

Given the retrospective nature of our analysis, it is not possible to assess the factors influencing the physicians’ and patients’ decision regarding treatment discontinuation. Potential sex and gender differences in decision making are insufficiently studied. Current guidelines on treatment duration are lacking for most cancer types, although data from retrospective cohorts and pooled subgroup analysis from clinical trials indicate that a minority of patients (in particular those with durable remission or irAEs) might benefit from treatment cessation (Marron et al. 2021). In fact, while initial clinical trials were designed for treatment until unacceptable toxicity or disease progression, most trials today limit treatment duration to 2 years. Although radiological response might be delayed and difficult to differentiate from (pseudo-)progression due to immune infiltration of the tumors, responses to ICI appear usually very early (Marron et al. 2021).

Recommendations based on the ESMO consensus conference suggest that considering treatment discontinuation is safe for melanoma patients presenting CR or PR or at least SD after a minimum treatment duration of 6 months (Keilholz et al. 2020). However, given the differences in tumor biology, the duration of treatment might depend on tumor type and patient characteristics as some patients might present durable responses even with minimal exposure to ICI. In addition, the arguments for and against treatment discontinuation should be thoroughly discussed with the patients as shared decision-making can increase the acceptance of adverse outcomes.

The overall frequency of irAEs occurring in our cohort (31.3%) was in the range of other real-world reports (Chen et al. 2021; Bastacky et al. 2021; Zheng et al. 2021). In agreement with previous publications (Unger et al. 2022; Duma et al. 2019), female sex was associated with higher risk of experiencing irAEs (38.4% versus 28.1%). Given the considerably higher incidence of irAEs in patient < 65 years as compare to the older age groups, the younger age of the female patients can partially explain this excess risk. Yet, several sex differences in immune responses, hormone levels and body composition as well as gender differences in reporting of irAEs might also contribute to these findings (Ozdemir and Dotto 2019). irAEs led to treatment discontinuation more often in females (21.1% vs. 16.8%). Since in clinical trials, irAEs as well as discontinuation rates are usually not reported by sex, a comparison of our results with clinical trials is difficult. It is conceivable that in real-world settings, rather high grade irAEs are registered in electronic health records while most low grade, self-limiting toxicities that do not require specific management or treatment discontinuation are omitted. The discontinuation rate could, therefore, be a good approximation for severe, clinically relevant irAEs.

The median time to onset of irAEs in our study (85 days) was longer than the 63 days reported by Ghisoni et al. for another Swiss Center on melanoma and NSCLC patients (Ghisoni et al. 2021). Both are however shorter than the median time to onset of 103 days (14.3 weeks) found in an analysis of 23 ICI trials including over 8400 patients between 2007 and 2019 (Tang et al. 2021). This may have been due to the increased awareness and earlier detection of irAEs in the recent years.

In our cohort the majority of the irAEs (61.5%) were managed in a multidisciplinary team with the implication of an organ specialist, indicating the adoption of international recommendations by the physicians. This multidisciplinary management is particularly important for the subset of patients, 8.3% in our cohort, who are steroid refractory and require additional immunosuppressive agents and close monitoring. In line with Ghisoni et al. who reported the rate of ongoing toxicity from the time of first toxicity onset to be 42.8, 38.4 and 35.7% at 6, 12 and 24 months, respectively (Ghisoni et al. 2021), in our cohort 38.8% of the patients received long-term hormonal substitution for ICI-related toxicities, illustrating the risk of irreversible irAEs.

Not surprisingly, patients with brain metastases at diagnosis had poor survival. Female patients had a longer OS than male patients in all age groups except among those older than 75 years, where males were observed to have a slight survival advantage. This is possibly related to the younger age of the female patients in our study, considering that patients younger than the median age of 66 years also had a longer OS. Certain meta-analyses (Conforti et al. 2018; Pinto et al. 2018) have suggested that female patients may benefit less from ICI treatment. However, it is important to consider that these studies were based on published hazard ratios for survival from different trials (not from individual patient data) that did not stratify by sex nor were powered to detect potential sex differences in efficacy. In addition to real-world analysis, reporting of toxicity and survival data of clinical trials by sex is required to fully understand the association between sex, age, irAEs and survival.

The OS of patients in our study presenting with any type and grade of irAEs was more than twice as long compared to those who did not experience any irAEs (3.1 versus 1.2 years), which is in line with findings from several meta-analyses as well as real-world reports in multiple cancer types (Fausto et al. 2021; Fan et al. 2021; Foster et al. 2021). It is possible that the higher incidence of irAEs in female patients results in longer survival. However, a causality cannot be presumed given that our findings may have been affected by lead-time bias.

Our analysis has some limitations. Since it is a retrospective, observational study, some clinical information was not available or incompletely collected (e.g., comorbidities, grading of irAEs). For instance, tumor response is rarely assessed and reported according to RECIST in clinical practice. In addition, the ECOG status was not systematically registered which limits the strength of our conclusions based on age and sex without taking into account the ECOG status.

Taken together, our findings indicate that ICI play an important role in oncology practice and have had a substantial increase in prescription rates over the past 5 years, even among the elderly and in patients with preexisting comorbidities. Female patients are at higher risk of experiencing irAEs and show longer OS than males. The sex- and gender-related factors contributing to these differences need to be explored.
